# Osteoporosis and hearing loss: findings from the Korea National Health and Nutrition Examination Survey 2009–2011^☆^

**DOI:** 10.1016/j.bjorl.2018.12.009

**Published:** 2019-02-18

**Authors:** Jun-Il Yoo, Ki Soo Park, Sung-Hyo Seo, Hyun Woo Park

**Affiliations:** a Gyeongsang National University Hospital, Department of Orthopaedic Surgery, Jinju, Republic of Korea; b Gyeongsang National University, Institute of Health Sciences, Jinju, Republic of Korea; c Gyeongsang National University School of Medicine, Department of Preventive medicine, Jinju, Republic of Korea; d Gyeongsang National University Hospital, Department of Otorhinolaryngology-Head and Neck Surgery, Jinju, Republic of Korea

**Keywords:** Osteoporosis, Femur neck, Age-related hearing impairment, Pure tone audiometry, Osteoporose, Colo do fêmur, Deficiência auditiva relacionada à idade, Audiometria tonal

## Abstract

**Introduction:**

Age-related hearing impairment is the most common sensory dysfunction in older adults. In osteoporosis, the mass of the ossicles will be decreased, affecting the bone density of the cochlea, and interfering with the sound transmission to the cochlea. Age related hearing loss might be closely related to osteoporosis.

**Objective:**

To determine the relationship between age-related hearing impairment and osteoporosis by investigating the relationship between hearing loss and cortical bone density evaluated from femur neck bone mineral density.

**Methods:**

We used data from the Korea National Health and Nutrition Examination Survey to examine the associations between osteoporosis and age-related hearing impairment from 2009 to 2011. Total number of participants was 4861 including 2273 men and 2588 women aged 50 years or older. Osteoporosis was defined as a bone mineral density 2.5 standard deviations below according to the World Health Organization diagnostic classification. Age-related hearing impairment was defined as the pure-tone averages of test frequencies 0.5, 1, 2, and 4 kHz at a threshold of 40 dB or higher on the more impaired hearing side.

**Results:**

Total femur T-score (*p* < 0.001), lumbar-spine T-score (*p* < 0.001) and, femur neck T-score (*p* < 0.001) were significantly lower in the osteoporosis group compared to the normal group. Thresholds of pure-tone averages were significantly different in normal compared to osteopenia, and osteoporosis groups. In addition, there were significantly higher pure-tone averages thresholds in the osteoporosis group compared to other groups (*p* < 0.001). After adjusting for all covariates, the odds ratio for hearing loss was significantly increased by 1.7 fold with reduced femur neck bone mineral density (*p* < 0.01). However, lumbar spine bone mineral density was not statistically associated with hearing loss (*p* = 0.22).

**Conclusion:**

Our results suggest that osteoporosis is significantly associated with a risk of hearing loss. In addition, femur neck bone mineral density was significantly correlated with hearing loss, but lumbar spine bone mineral density was not.

## Resumo

**Introdução:**

A perda auditiva associada ao envelhecimento é a disfunção sensorial mais comum em idosos. Na osteoporose, a massa dos ossículos diminui e afeta a densidade óssea da cóclea, o que irá interferir na transmissão do som para a mesma. A perda auditiva associada à idade pode estar intimamente relacionada à osteoporose.

**Objetivo:**

Determinar a relação entre deficiência auditiva relacionada à idade e osteoporose, investigar a relação entre perda auditiva e densidade óssea cortical avaliada a partir da densidade mineral óssea do colo do fêmur.

**Método:**

Utilizamos dados da *Korea National Health and Nutrition Examination Survey* para examinar as associações entre osteoporose e perda auditiva associada ao envelhecimento de 2009 a 2011. O número total de participantes foi de 4.861, incluiu 2.273 homens e 2.588 mulheres com 50 anos ou mais. A osteoporose foi definida como densidade mineral óssea com 2,5 desvios-padrão abaixo da média, de acordo com a classificação diagnóstica da Organização Mundial da Saúde. A perda auditiva associada ao envelhecimento foi definida como as médias de tom puro das frequências de teste de 0,5, 1, 2 e 4 kHz a um limiar de 40 dB ou superior no lado da audição mais afetado.

**Resultados:**

O T-score total do fêmur (*p* < 0,001), o T-score da coluna lombar (*p* < 0,001) e o T-score do colo do fêmur (*p* < 0,001) foram significantemente menores no grupo com osteoporose em comparação ao grupo normal. Os limiares de médias de tom puro foram significantemente diferentes nos grupos normais em comparação com aqueles com osteopenia e osteoporose. Além disso, houve limiares significantemente maiores de médias de tom puro no grupo com osteoporose em comparação com os outros grupos (*p* < 0,001). Após o ajuste para todas as covariáveis, a *odds ratio* da perda auditiva mostrou estar significantemente aumentada em 1,7 vez com densidade mineral óssea reduzida no colo do fêmur (*p* < 0,01). No entanto, a densidade mineral óssea da coluna L não se associou estatisticamente à perda auditiva (*p* = 0,22).

**Conclusão:**

Nossos resultados sugerem que a osteoporose está significantemente associada ao risco de perda auditiva. Além disso, a densidade mineral óssea da coluna lombar não se correlacionou com a perda auditiva, apenas a densidade mineral óssea do colo do fêmur foi significantemente correlacionada.

## Introduction

As population aging is occurring in many Countries, the importance of health life expectancy of elderly people is becoming a major concern. Furthermore, there is a growing interest in chronic and aging diseases that affect the health expectancy of elderly people.^1^, ^2^, ^3^ Of these aging diseases, age-related hearing impairment (ARHI) is the most common sensory dysfunction in older adults. ARHI reduces the quality of life for the elderly and makes communication difficult often with resulting social isolation.^4^, ^5^, ^6^ Several studies have suggested the risk factors for ARHI include traditional cardiovascular risk factors, such as hypertension, chronic kidney disease, and diabetes mellitus.^7^, ^8^, ^9^, ^10^ As a result, it appears that systemic conditions can affect hearing loss.

Recently, Yeh et al.^11^ performed the largest population-based study to evaluate the risk of sudden sensorineural hearing loss (SSNHL) in a national cohort of Asian patients with osteoporosis. They reported a 1.76 fold increase in the incidence of SSNHL for patients with osteoporosis compared with the comparison group after covariates such as age, sex, medical comorbidities, geographical area, and monthly income were considered. In addition, they suggested that demineralization of the cochlear capsule was found to be correlated with hearing loss in patients with metabolic bone disorders. However, another study investigating 120 postmenopausal women, showed no statistical significance at low frequencies, irrespective of bone mineral density (BMD) values.^12^

Zehnder et al., investigating bone metabolism in the otic capsule, suggested that OPG, a potent inhibitor of osteoclasts, is present in the inner ear and is secreted as perilymph to inhibit bone remodeling of the otic capsule, and in particular the cochlea.^13^ Kanzaki et al. demonstrated that various parts of ossicles were thinned and weakened and that the ligaments between the stapes and the oval window also disappeared. In hearing threshold studied by acoustic brainstem response, the overall hearing threshold was higher in the Opg –/– mouse model than in the normal group as age increased. In particular, the hearing threshold of 20 dB or more was observed in the high frequency above 20 kHz. However, they could not confirm which ossicles were more vulnerable.^14^

When the sound is presented to the external ear canal, it is transmitted through the ossicles vibration to the cochlea. That ossicular vibration is directly proportional to the stiffness of the tympanic membrane, inter-ossicular joint and oval window, and inversely proportional to the mass of the tympanic membrane and ossicles.^15^, ^16^ Mass helps to transmit low frequency vibration and prejudice the transmission of high-frequency sounds, while stiffness helps high-frequency vibration and disturbs low-frequencies transmission. Various middle ear pathologies can affect the sound transmission as vibration to the inner ear by changing the mass of the ossicles and the stiffness of the middle ear. Examples of pathologies associated with increased stiffness include negative middle ear pressure, otosclerosis, and otitis media. Definite example of decreased stiffness is an ossicular disruption, when vibration cannot be transmitted to the cochlea. In osteoporosis, the mass of the ossicles will be decreased, affecting the bone density of the cochlea, which will interfere with the sound transmission to the cochlea.^15^, ^17^

Our hypothesis was that age related hearing loss could be closely related to osteoporosis, and that BMD levels at the femur neck, which occupies a large portion of the cortical bone, would reflect the condition more than the BMD lumbar spine levels.

Therefore, the purpose of this study was to determine the relationship between ARHI and osteoporosis and to investigate the relationship between cortical bone density evaluated from lumbar spine (L-spine) and femur neck BMD, and hearing loss.

## Methods

### Ethics statement

Data from the 2009–2011 Korean National Health and Nutrition Examination Survey (KNHANES) was reviewed and approved by the Institutional Review Board of the Korea Centers for Disease Control and Prevention (KCDC) (Approval no. 2009-01CON-03-2C, 2010-02CON-21-C, and 2011-02CON-06-C). Written informed consent was obtained from all participants when the 2009, 2010, and 2011 KNHANES were conducted.

### Study population

**Figure 1:**
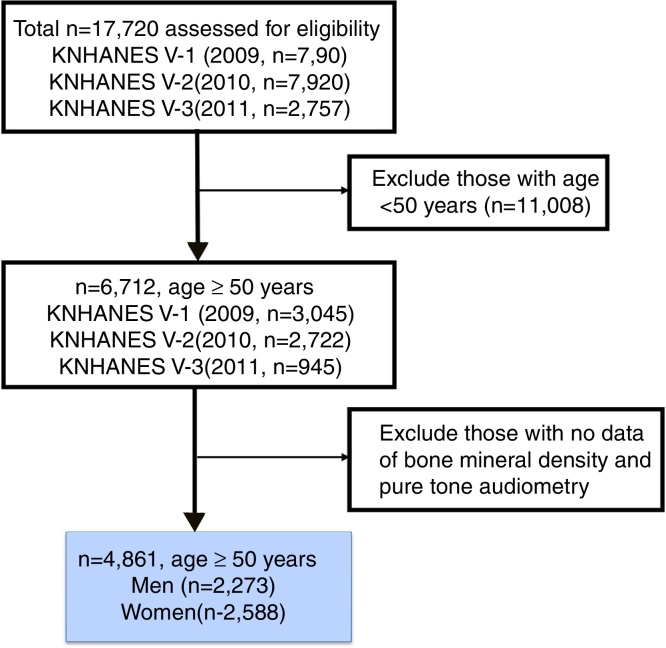
Flow sheet of study participants.

KNHANES has been a nationwide representative cross-sectional survey for the Korean population with a clustered, multistage, stratified, and rolling sampling design. KNHANES consists of a health interview, health examination, and dietary survey. The survey data is collected from household interviews and direct standardized physical examinations conducted in specially equipped mobile examination centers. The data was collected from 17,720 participants in 2009 (*n* = 7920), 2010 (*n* = 7043), and 2011 (*n* = 2757). Patients under 50 years of age and with no registered data on bone mineral density or pure tone audiometry were excluded. After these exclusions, a total of 4861 participants (2273 men and 2588 women) with normal tympanic membrane were analyzed (Fig. 1).

### Assessment of osteoporosis

Bone Mineral Content (BMC) and Bone Mineral Density (BMD) from total femur, and from femoral neck and lumbar spine (L1–L4) were measured by trained technicians using DXA (QDR 4500A, Hologic Inc., Waltham, MA, USA). Osteopenia or osteoporosis were diagnosed with supplying by the DXA manufacturer^18^ using T-score of the total femur, femoral neck, and lumbar spine according to the criteria of the World Health Organization (T-score ≥ −1; normal, −2.5 < T-score < −1; osteopenia, T-score ≤ −2.5; osteoporosis).^19^

### Assessment of hearing threshold

For evaluation of hearing threshold, pure-tone audiometry was conducted with a SA 203 audiometer (Entomed; Malmö, Sweden) in a soundproof booth and instructions were given by a trained otolaryngologist. In a soundproof booth, subjects put on a headset, and pushed a button when they heard a pure tone sound. Only air conduction threshold was measured. The pure-tone averages (PTA) were calculated as the average of threshold at 0.5, 1, 2 and 4 kHz. Hearing loss was defined as the PTA of threshold 40 dB or higher.

### Assessment of covariates

Information on the study population including BMI, smoking, alcohol consumption, physical activity, and medical conditions were obtained from KNHANES data. BMI was calculated from the height which was measured to the nearest 0.1 cm with a stadiometer.

Hypertension was defined as a systolic Blood Pressure (BP) of 140 mmHg, a diastolic BP of 90 mmHg, or treatment with antihypertensive agents. Diabetes mellitus was defined by a fasting plasma sugar level >126 mg/dL, treatment with oral hypoglycemic agents or insulin, or diagnosis by a physician.

In terms of smoking, the participants were categorized either as current smokers or others. A participant was considered to have a “monthly alcohol ingestion history” if he/she drank more than once per month over the past year. A moderate physical activity done for at least 20 min a time and more than three times a week was considered to be regular exercise.

### Biochemical analysis

Serum 25 (OH)D and parathyroid hormone (PTH) levels were measured using a gamma counter (1470 Wizard; Perkin Elmer, Turku, Finland) and LIAISON (DiaSorin) with radioimmunoassay (25 (OH)D 125I RIA Kit; DiaSorin) and chemiluminescence immunoassay (N-tact PTH Assay kit; DiaSorin), respectively.

### Statistical analysis

To compare PTA by presence of osteoporosis, ANOVA with Bonferroni correction was performed. Multiple logistic regression analyses were also performed to estimate the association between T-score and hearing loss. Data are presented as OR (95% CI) after adjusting for certain factors in each model using hierarchical analysis (Model 1: adjusted for age; Model 2: adjusted for age, current smoking, alcohol use, regular exercise, and body fat percentage; Model 3: Adjusted for age, current smoking, alcohol use, regular exercise).

To reflect the sampling weights, complex sampling analyses was performed. All statistical analyses were performed using the SAS (version 9.3; SAS Institute, Cary, NC, USA). All data with *p* < 0.05 were accepted as statistically significant results.

## Results

Subject characteristics are shown in Table 1 (*p* < 0.001). Osteopenia and osteoporosis were more common in women than men. Current smoking (*p* < 0.001), monthly alcohol drinking (*p* < 0.001), BMI (*p* < 0.001) were significantly higher in the normal group compared to the osteopenia and osteoporosis group. However, moderate physical activity, diabetes mellitus, and hypercholesterolemia were not significantly different.

**Table 1:** Demographics and clinical characteristics of the study population.

Variable	Normal	Osteopenia	Osteoporosis	*p*-value
	*n* = 1299	*n* = 2388	*n* = 1174	
Age (years)	60.19 ± 7.74	63.29 ± 8.38	69.25 ± 8.48	<0.001
Male gender (%)	957 (73.67)	1109 (46.44)	207 (17.63)	<0.001
BMI (kg/m^2^, mean ± SD)	24.93 ± 2.79	23.92 ± 3.07	23.04 ± 3.09	<0.001
Current smoker (yes, %)	293 (22.61)	417 (17.58)	128 (11.01)	<0.001
Monthly alcohol history (yes, %)	816 (63.16)	1041 (44.02)	285 (24.57)	<0.001
Moderate physical activity (%)	164 (12.67)	291 (12.26)	131 (11.26)	0.544
Hypertension (%)	514 (39.63)	936 (39.36)	520 (44.41)	0.012
Diabetes mellitus (%)	215 (16.58)	357 (15.01)	158 (13.49)	0.161
Hypercholesterolemia (%)	253 (19.51)	428 (18.00)	163 (13.92)	0.003
L-spine T-score (mean ± SD)	0.20 ± 0.92	−1.37 ± 0.76	−2.88 ± 0.76	<0.001
Femur neck T-score (mean ± SD)	−0.11 ± 0.67	−1.39 ± 0.61	−2.48 ± 0.74	<0.001
Total femur T-score (mean ± SD)	0.62 ± 0.68	−0.50 ± 0.64	−1.52 ± 0.78	<0.001
Phosphorus intake (mg)	1289.58 ± 511.87	1116 ± 495.88	910.59 ± 423.39	<0.001
Calcium intake (mg)	582.86 ± 384.52	492.59 ± 335.33	381.07 ± 276.09	<0.001
Serum creatinine (mg/dL)	0.91 ± 0.23	0.83 ± 0.20	0.77 ± 0.26	<0.001
Vitamin D (ng/mL)	20.24 ± 6.76	19.64 ± 7.24	18.80 ± 7.28	<0.001
ALP (U/L)	229.73 ± 72.62	249.65 ± 71.14	268.85 ± 82.42	<0.001
PTH (pg/mL)	65.67 ± 24.69	68.0 ± 27.86	73.96 ± 41.14	<0.001

BMI, body mass index; ALP, alkaline phosphatase; PTH, parathyroid hormone.

L-spine T-score (*p* < 0.001), femur neck T-score (*p* < 0.001) and, total femur T-score (*p* < 0.001) were all significantly decreased in the osteoporosis group compared to controls. Calcium (*p* < 0.001), phosphorus intake (*p* < 0.001), serum creatinine (*p* < 0.001), and vitamin D (*p* < 0.001) were also significantly decreased in the osteoporosis group. However, alkaline phosphatase *p* < 0.001) and parathyroid hormone (*p* < 0.001) were significantly increased in the osteoporotic group.

**Figure 2:**
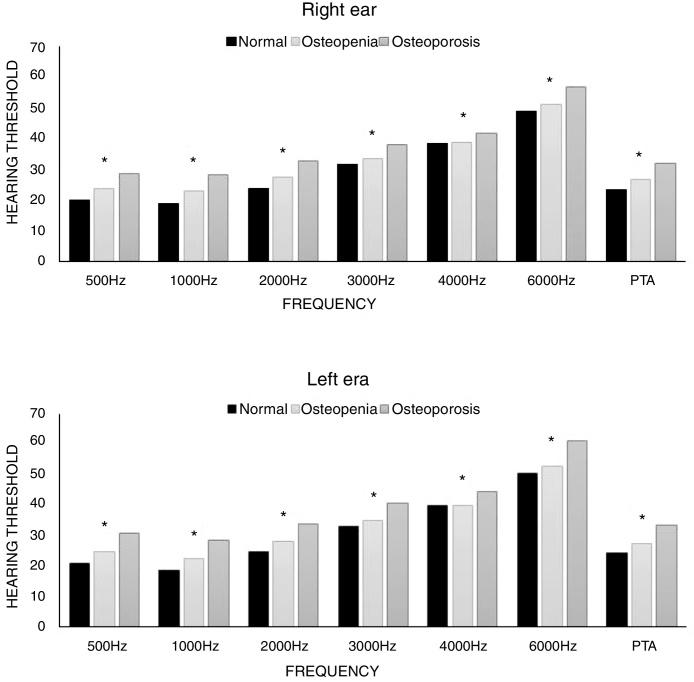
Correlation between hearing threshold and frequency of each group. PTA, pure tone average; average threshold at 500, 1000, 2000, 4000 Hz.

Thresholds of PTA were significantly different between the normal and osteopenia, and osteoporosis groups. Significantly higher PTA threshold was seen in the osteoporosis group compared to the other groups (*p* < 0.001) (Fig. 2, Supplement 1).

After adjustment for all covariates, such as the age, gender, hypertension, phosphorous intake, serum creatinine, vitamin D, hypercholesterolemia, and monthly alcohol history, the Odds Ratio for hearing loss were significantly increased 1.7 fold with decreasing femur neck BMD (*p* < 0.01). However, L-spine and femur shaft BMD were not statistically correlated with hearing loss, respectively (*p* = 0.22 and 0.16) (Table 2).

**Table 2:** Logistic regression analysis for bone mineral density by presence of hearing loss.

Variables	OR	95% Wald	*p*-value
Femur neck BMD^a^	1.655	1.491–1.837	<0.01
Femur shaft BMD^a^	0.922	0.821–1.034	0.16
L-spine BMD^a^	0.953	0.883–1.029	0.22

BMD, bone mineral density.

a Adjusted covariates such as age, gender, hypertension, phosphorous intake, serum creatinine, vitamin D, hypercholesterolemia, and monthly alcohol history.

## Discussion

The principle finding of the present study is that there is a significant correlation between osteoporosis and ARHI. Although L-spine BMD and hearing loss were not significantly related, femur neck BMD and hearing loss were significantly related.

Several studies have reported an association between osteoporosis and hearing loss in the older population. However, this relationship is controversial. Jung et al. performed a case-control study using 1009 postmenopausal women. They reported no association between bone mineral density and hearing impairment in the study population.^20^ Nevertheless, a number of other studies report that ARHI and osteoporosis are related. Recently, Ye et al. investigated hearing loss in the Taiwanese health insurance database from 1998 to 2008 with 16,600 cases diagnosed with osteoporosis and 30,080 cases without osteoporosis. They found that the relative risk of hearing loss in the osteoporotic group was 1.76 times higher than in the control group, as a result of matching age, sex, diabetes, hypertension, cardiovascular disease, and chronic kidney disease. Our study, in close agreement with these findings, showed that the odds ratio of hearing loss in the osteoporotic group was 1.7 times higher. However, we believe that our data is more objective due to the use of pure tone threshold compared to their analysis in which the diagnostic code was used.

In this study, L-spine BMD was not associated with hearing loss. Seventy five percent of L-spines are composed of trabecular bone while 75% of the femur neck are composed of cortical bone.^21^ Since the configuration of the proximal femur is similar to that of ossicles, especially the malleus, and as cavitation of bone increases in osteoporosis, a similar phenomenon occurs in the ossicles. Therefore, it seems reasonable that the correlation will be more significant in the femur neck than in the femur shaft and L-spine.^22^ A previous study reported an association between BMD and hearing loss in postmenopausal patients.^12^ Although the mechanism of hearing loss is unclear, it has been suggested that demineralization of the otic capsule is associated with secondary neuronal degeneration, resulting in sensorineural hearing loss.^23^, ^24^

There were several limitations to this study. First, it was a cross-sectional study and retrospective in design. Therefore, we could not evaluate the causality between bone mineral density and hearing loss. Prospectively designed studies are necessary to clarify this relationship. Second, there is a lack of explanation for the mechanism of action. Therefore, well-designed experimental studies will be necessary to clarify this mechanism. Third, we could not reflect mild hearing loss and young adults. Therefore, in the future, large-scale studies including mild hearing loss and young adults will be necessary. Finally, there are differences in biochemical factors in east Asian that can affect osteoporosis and hearing loss compared to that in Western countries. In particular, the proportion of vitamin D deficiency in elderly people is higher in Korea than in Western countries. However, in this study, biochemical factors including vitamin D were adjusted for statistical analysis.

## Conclusion

Osteoporosis is significantly associated with a risk of hearing loss. In addition, lumbar spine bone mineral density al density was not correlated with hearing loss, while femur neck bone mineral density was significantly correlated.

## Funding

This study was funded by the Ministry of SMEs and Startups, Republic of Korea (Project No. P0002726).

The funder had no role in study design, data collection and analysis, decision to publish, or preparation of the manuscript.

## Conflicts of interest

The authors declare no conflicts of interest.
