# Supplementary material for: Osteoporosis and hearing loss: findings from the Korea National Health and Nutrition Examination Survey 2009–2011
Source: Braz J Otorhinolaryngol. 2019 Feb 18;86(3):332–8. doi: 10.1016/j.bjorl.2018.12.009 (PMC9422524; doi:10.1016/j.bjorl.2018.12.009)
Supplement: Supplementary file 1 [file mmc1.docx]

Supplement table 1. Correlation between hearing threshold and frequency of each group

|  | **Frequency** | **Normal (1,299)** | | **Osteopenia (2,388)** | | **Osteoporosis (1,174)** | | **P** | **P1** | **P2** | **P3** |
| --- | --- | --- | --- | --- | --- | --- | --- | --- | --- | --- | --- |
|  |  | Mean | SD | Mean | SD | Mean | SD |  |  |  |  |
| Right | 500Hz | 19.87 | ±17.71 | 23.62 | ±19.55 | 28.72 | ±21.85 | <0.001 | <0.001 | <0.001 | <0.001 |
|  | 1000Hz | 18.90 | ±17.91 | 22.82 | ±19.90 | 28.21 | ±22.70 | <0.001 | <0.001 | <0.001 | <0.001 |
|  | 2000Hz | 23.56 | ±19.63 | 27.25 | ±20.64 | 32.53 | ±22.53 | <0.001 | <0.001 | <0.001 | <0.001 |
|  | 3000Hz | 31.45 | ±23.30 | 33.47 | ±23.50 | 37.84 | ±23.78 | <0.001 | 0.039 | <0.001 | <0.001 |
|  | 4000Hz | 38.33 | ±24.78 | 38.91 | ±25.08 | 41.70 | ±24.68 | 0.001 | 1 | 0.0023 | 0.005 |
|  | 6000Hz | 48.80 | ±25.14 | 51.08 | ±25.80 | 56.85 | ±25.09 | <0.001 | 0.0275 | <0.001 | <0.001 |
|  | **PTA** | **23.45** | **±17.63** | **26.79** | **±19.06** | **31.82** | **±21.11** | **<0.001** | <0.001 | <0.001 | <0.001 |
| Left | 500Hz | 20.75 | ±17.27 | 24.48 | ±19.15 | 30.87 | ±21.74 | <0.001 | <0.001 | <0.001 | <0.001 |
|  | 1000Hz | 18.53 | ±18.05 | 22.35 | ±19.82 | 28.60 | ±22.80 | <0.001 | <0.001 | <0.001 | <0.001 |
|  | 2000Hz | 24.46 | ±20.43 | 27.90 | ±21.47 | 33.58 | ±22.88 | <0.001 | <0.001 | <0.001 | <0.001 |
|  | 3000Hz | 33.06 | ±23.98 | 34.74 | ±23.94 | 40.43 | ±24.06 | <0.001 | <0.001 | <0.001 | <0.001 |
|  | 4000Hz | 39.76 | ±25.04 | 39.70 | ±25.20 | 44.61 | ±24.47 | <0.001 | 1 | <0.001 | <0.001 |
|  | 6000Hz | 50.70 | ±25.37 | 52.98 | ±25.60 | 61.05 | ±25.44 | <0.001 | 0.0282 | <0.001 | <0.001 |
|  | **PTA** | **24.20** | **±17.84** | **27.37** | **±19.36** | **33.37** | **±21.29** | **<0.001** | <0.001 | <0.001 | <0.001 |

PTA: Pure tone average; average threshold at 500, 1000, 2000,4000Hz; P1: P-values for differences in Normal and Osteopenia ; P2: P-values for differences in Normal and Osteoporosis ; P1: P-values for differences in Osteopenia and Osteoporosis
